# Water Film-Mediated Hydrolysis of Pyrophosphate on a Nanosized Mn Oxide

**DOI:** 10.1021/acs.langmuir.5c02476

**Published:** 2025-08-29

**Authors:** Tao Chen, Tao Luo, Tra My Bui Thi, Khalil Hanna, Jean-François Boily

**Affiliations:** † Department of Chemistry, 105361 Umeå University, Umeå SE-901 87, Sweden; ‡ Univ. Rennes, École Nationale Supérieure de Chimie de Rennes, CNRS, ISCR-UMR 6226, Rennes F-35000, France

## Abstract

The geochemical behaviors of phosphate-containing species at mineral surfaces are of fundamental importance for controlling phosphorus (P) mobility, fate, and bioavailability. Understanding these interfacial behaviors in water-unsaturated environments, where minerals are covered by thin water films, is of special importance in the context of soil vadose zone geochemistry. This study resolved the transformation of pyrophosphate to orthophosphate within the confines of nanometer-thick water films condensed on nanosized birnessite (MnO_2_). Time-resolved vibrational spectroscopy indicated that PP was effectively degraded into monodentate mononuclear complexes on MnO_2_ and at rates that followed zero-order kinetics. We found a direct link between PP degradation kinetics and water film thickness, which revealed the role of sorbed water as the key nucleophilic agent needed for P–O–P bond rupture. By highlighting the essential roles of surface complexation and water loadings in controlling pyrophosphate transformation, this work adds new insight needed to advance knowledge on the global cycling of P in nature. It can also have broader applications to catalysis and energy storage solutions involving MnO_2_ nanoparticles.

## Graphical abstract

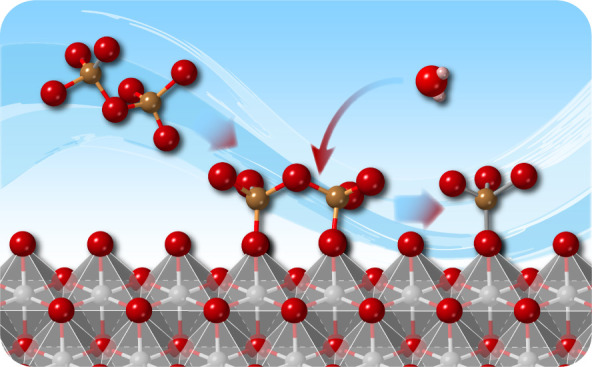

## Introduction

Phosphorus is an essential nutrient for all living organisms, and is present in various forms in nature.^1,2^ This includes polyphosphates, which is a group of phosphate-bearing compounds joined by phosphoanhydride (P–O–P) bonds. Polyphosphates represent important fractions of the total P in planktonic organisms (1 – 13%),^3,4^ dissolved and particulate pools of seawater (0 – 8%),^5,6^ marine sediments (0 – 8%),^7^ lake sediments (1.5 – 11.4%),^8^ and soils (0.4 – 7%).^9^ They can also be produced biogenically^10^ (e.g., extracellular release, lysis, and death) as well as by industrial discharge (e.g., water treatment processes, medicines, fertilizers, flame retardants, food additives).^11^ The biogenic and anthropogenic release of dissolved and particulate polyphosphates in natural watersheds (e.g., lakes, seawater, and sediments)^12^ calls for a new understanding of their fate and stability in nature. This knowledge has important implications in the study of geological and anthropogenic P cycles.^13–15^

Hydrolysis reactions of polyphosphates to orthophosphate (orthoP) are of great interest in this context. Other than the well-recognized enzymatic dephosphorylation by phosphatases,^7,16,17^ this process can be abiotically facilitated by extremely acidic^18,19^ or basic^20^ media. The nucleophilic attack of H_2_O or OH^–^ on the activated P–O–P bonds is one of the key steps toward bond cleavage,^21,22^ and therefore the production of shorter-chained polyphosphates:

1 $$H_{n+2}P_{n}O_{3n+1}+H_{2}O⇌H_{n+1}P_{n-1}O_{3n-2}+H_{3}{\mathrm{PO}}_{4}$$

However, they can also be catalyzed under circumneutral conditions through Lewis acid/leaving group activation by complexation with divalent metal cations^23^ or with metal ion centers at mineral surfaces (e.g., Mn-,^24^ Fe-,^25,26^ and Al-oxides.^13,27^ By relaxing local P–O–P bonds, complexation facilitates attacking by weak nucleophiles, water^18,24^ or hydroxyl groups,^22,28^ also giving way to bond cleavage.^21,28^ It is consequently possible that polyphosphate hydrolysis could be sensitive to water availability and therefore to air moisture, such as in water-unsaturated environments, including vadose zones of soils.^26^ This becomes especially important considering that minerals capture air moisture in the form of thin water films, whose thickness is directly affected by natural variations in humidity.^29,30^

This study uncovered the roles that air moisture plays in the hydrolysis of pyrophosphate (P_2_O_7_^4–^, PP) associated with birnessite (MnO_2_), a highly reactive and abundant Mn­(IV)-oxide nanomineral in nature.^31^ Focus on this simpler polyphosphate, which is present in nature^32,33^ at up to hundreds of μmol/L,^33,34^ provides an opportunity to follow the very last step in polyphosphate degradation into two orthoP species,^35^ a process that has received less attention in the scientific literature. Monitoring these reactions was made possible by *in situ* vibrational spectroscopy, which uncovered a direct link between PP degradation kinetics and water loadings. This study deepens our understanding of polyphosphate degradation by exploring the water film-driven decomposition of PP in terms of surface complexation and activation reactions. This knowledge can be directly tied to processes in vadose zones of soils,^36^ and contributes to the understanding of P cycling^1^ and primary production in ecosystems.^37^ It can also have implications for catalysis and energy storage solutions involving MnO_2_.

## Experimental Section

### Materials

Stock solutions of sodium orthophosphate (Na_3_PO_4_) and sodium pyrophosphate decahydrate (Na_4_P_2_O_7_·10H_2_O) (Sigma-Aldrich) were prepared in ultrapure water. Standard solutions of premade 1 M NaOH or 1 M HCl (Sigma-Aldrich) were used to adjust the pH of the solutions. Acid birnessite was synthesized by reacting KMnO_4_ and HCl in boiling water.^31,38^ The resulting suspension (26 g/L) was purged from dissolved atmospheric CO_2_(g) using a stream of N_2_(g) for 2 h, then stored in the dark at 4 °C. The study of Li et al.^31^ contains a detailed report on the synthesis and purification procedures, as well as salient physicochemical properties of the high specific surface area (85 m^2^/g) MnO_2_ nanoparticles measured by Brunauer–Emmett–Teller (BET) N_2_ adsorption.

### PP Degradation in Water Films on Nanosized MnO_2_

Vibrational spectroscopy was used for *in situ* tracking of water film populations and surface species transformation on dry MnO_2_ coatings exposed to humid air at 25 °C. MnO_2_ coatings were obtained by pipetting a 5 mL droplet of 2.6 g/L MnO_2_ suspension on a thermostatic optical diamond window of an Attenuated Total Reflection (ATR) accessory (Golden Gate, diamond single bounce N29328, Specac) in a closed chamber. The wet MnO_2_ coatings were dried under a flow of 500 mL/min dry N_2_(g) (∼ 0% RH, 0.0095 kPa H_2_O). Continuous monitoring of water O–H stretching and H–O–H bending bands indicated that the sample lost all free water within 20 min.

A 5 mL aliquot of a 2 mM PP solution adjusted to pH 7 was thereafter cast onto the dried MnO_2_ coating. The resulting sample was then dried under a stream of dry N_2_(g) for no more than 20 min. Note that because PP hydrolysis is a slow process operating over the course of hours, it is unlikely that this initial sample preparation step generated any orthoP. PP hydrolysis reactions were thereafter monitored by continuously acquiring spectra as the samples were exposed to a stream of moist N_2_(g) at 10, 30, 60, 80, and 95% RH. Gas composition was generated and controlled using a humidity generator module (proUmid MHG32).

All vibrational spectra were collected on samples prepared on the ATR cell using a Fourier Transform Infrared (FTIR) spectrometer (Bruker, Vertex 70/v). Measurements were acquired in the 600–4500 cm^–1^ range at a resolution of 4 cm^–1^, and at a forward/reverse scanning rate of 10 kHz. Spectra were generated with a Blackman-Harris 3-term apodization function with 16 cm^–1^ phase resolution and the Mertz phase correction algorithm. Each spectrum was obtained by coadding 200 spectra, which were collected over a 178 s period.

### PP and orthoP Sorption Experiments

Effects of surface loadings on the vibrational spectra of PP and orthoP on MnO_2_ surfaces were measured to assist in the interpretation of the hydrolysis experiments. Those for PP were conducted in the same manner as in the degradation experiments, but only the starting spectra were collected. Dry MnO_2_ coatings previously prepared on the ATR cell were treated with 5 mL droplets of 1, 2, 5, and 10 mM PP solutions at pH 7. The samples were dried under N_2_(g), and ATR-FTIR spectra were then collected in 20 min as the samples were exposed to a stream of 80% RH. PP loadings were obtained by batch adsorption. Suspensions of 2.6 g/L MnO_2_ were mixed with 1, 2, 5, 10 mM PP solutions, and the mixtures were adjusted to pH 7 using 1 M NaOH or HCl. The resulting suspensions were equilibrated at pH 7 for 24 h at 25 °C on an end-to-end rotator in the dark.

Surface loadings of orthoP were studied by batch adsorption in the same fashion. Here orthoP solutions at concentrations of 0.5, 1, 5, 10, and 20 mM were each mixed with a 1 g/L suspension of MnO_2_. The resulting mixtures were then adjusted and equilibrated at pH 7 for 24 h. The effects of pH on orthoP adsorption mechanisms were also investigated by reacting 1 mM orthoP solutions with 1 g/L MnO_2_ at pH 4, 7, and 9 for 24 h. The centrifuged wet pastes of the resulting suspensions were applied on the ATR cell using a spatula, and dried under N_2_(g). ATR-FTIR spectra were thereafter collected as the dried pastes were exposed to 80% RH.

The resulting supernatants above were filtered (0.22 μm hydrophilic polytetrafluoroethylene, H-PTFE-20/13, Duren-Germany), and concentrations of PP and orthoP in the filtrate were measured by Ion Chromatography (IC). The IC instrument (DX-120) was operated with an anion separation column (Dionex IonPac AS19 – 4 × 250 mm) coupled with a conductivity detector. Elution was performed at a flow rate of 1 mL/min of deionized water. Surface loadings of PP (Γ_PP_) and orthoP (Γ_orthoP_) on MnO_2_ were then calculated from the difference between the initial and final concentrations.

### Water Loadings by Microgravimetry and Vibrational Spectroscopy

Water loadings on the synthetic MnO_2_ powder were determined by dynamic vapor sorption (DVS), using a DVS Advantage ET 2 instrument (Surface Measurement systems). These were obtained through an 11-point adsorption isotherm between 0 and 95% RH (0.0095 – 3.011 kPa H_2_O) at 25 °C. Measurements were made using ∼ 30 mg samples initially dried at ∼ 0% RH (25 °C) for 10 h to remove adsorbed water. The samples were thereafter exposed to a flow of moist N_2_(g) at preselected levels of RH. Equilibration times of 60 min for samples exposed up to 60% RH were extended to 180 min at higher RH to allow masses to reach time-independent values before each adsorption step. Gravimetric measurements were continuously taken every 1 min, and a complete adsorption isotherm took up to 28 h. The gravimetrically determined water loadings were expressed in terms of H_2_O/nm^2^, using 85 m^2^/g as the specific surface area of MnO_2_. The number of equivalent water monolayer (ML) was estimated assuming that 1 ML contains 12 H_2_O/nm^2^, which is a typical value of water films on (hydr)­oxo-terminated metal oxide surfaces.^39,40^

ATR-FTIR spectra of water films formed on PP-free and PP-bearing MnO_2_ were also collected at 0 – 95% RH, using the same experimental setup as in the PP hydrolysis experiments. Equilibration times were the same as in the microgravimetric measurements.

### Vibrational Spectral Analysis

The time-resolved PP degradation spectra were analyzed by (i) Multivariate Curve Resolution (MCR)^41^ and (ii) Gaussian deconvolution. All procedures were performed on spectra with absorbances offset to zero at (i) 1250 cm^–1^ for the P–O­(H) stretching region (850–1250 cm^–1^),^15^ (ii) 1800 cm^–1^ for the water bending region (1300–1800 cm^–1^), and (iii) 4000 cm^–1^ for the O–H stretching region (2500–4000 cm^–1^). All vibrational spectra were normalized to the Mn–O stretching band intensity at 600 cm^–1^ to account for variations in sample quantity prior to the analysis.

The ATR-FTIR spectra collected in water sorption experiments on the PP-free and PP-bearing MnO_2_ were also analyzed by MCR to relate MCR concentration profiles to water loadings. More details on this procedure are described in the Supporting Information.

MCR extracted spectral profiles and their relative concentrations of end-member components. This was achieved by expressing the matrix (**A**) of time-resolved spectra in terms of a linear combination of the product of the spectral (**ε**) and concentration (**C**) profiles, which is related through the Beer–Lambert law (**A** = **εC**). This was achieved using the MCR-ALS program^41^ in the computational environment of MATLAB (The Mathworks, Inc.). Gaussian deconvolution was performed on the same dataset, however corrected by a linear background. The resulting datasets (**A’**) were deconvoluted with

2 $$A{\left(n\right)}^{\prime }=\sum_{1}^{n}A{\left(n\right)}_{n,\max}^{\prime }e^{-{\left(\left(v-v_{n}\right)/\sigma_{n}\right)}^{2}}$$

where *A*’_*n*,max_ is the maximal absorbance of the *n*th component centered at wavenumber *v_n_* with the width distribution σ*_n_*.

### Surface and Solid Characterizations

To probe the surface state of the mixtures in the PP degradation experiments, MnO_2_ layers were cast and dried on glass slides by pipetting 200 mL of a suspension of 2.6 g/L MnO_2_. Another 200 mL of a 2 mM PP solution at pH 7 was then cast over the dried MnO_2_ layers. The mixtures were dried and exposed to a stream of 80% RH in N_2_(g), and the solids were collected after reaction of 0, 1, 2, and 7 days. The surface composition of the samples was analyzed using X-ray photoelectron spectroscopy (XPS). The instrument was a Kratos Axis Ultra electron spectrometer, equipped with a monochromatic Al *K*_α_ X-ray (1486.7 eV) source operating at 10 mA and 15 kV. Our analysis was focused on the O 1s region to resolve the bonding environment of P-bound O. Reference spectra of sodium pyrophosphate decahydrate salt powder and dried pristine MnO_2_ were also acquired for comparison.

To exclude possible surface PP/orthoP precipitation or intercalation into MnO_2_ interlayers, the reacted solids were also investigated using X-ray diffraction (XRD). XRD patterns of pristine and reacted PP-loaded MnO_2_ were acquired on a Bruker D8 Advance diffractometer equipped with a copper anode tube (Cu *K*_α_ radiation). All XRD data were recorded in the 2θ = 5–80° range at an angular step size of 0.006°. Slightly different from the XPS measurements, pH 7 solutions of 1, 2, 5, and 10 mM PP were mixed with MnO_2_ coated on a low-background silica (Si) sample holder. The resulting mixtures were then measured immediately after a 20 min period of drying. Reference patterns of pure MnO_2_ were obtained following the same procedure. The sample prepared with 2 mM PP was, additionally, reacted *in situ* on the Si holder for 0, 1, and 7 days under a stream of 80% RH at 25 °C.

## Results and Discussion

The inherently low nucleophilicity of water, combined with a high activation energy barrier,^18,21,42^ makes the hydrolysis of PP (H_4_P_2_O_7_ + H_2_O ⇌ 2H_3_PO_4_) kinetically sluggish in circumneutral aqueous media.^43^ To assess how PP hydrolyzes in thin MnO_2_ water films under these conditions, we began by tracking the vibrational P–O stretching and P–O–H bending of PP as samples were exposed to a stream of 80% RH (Figure 1). The spectra initially revealed characteristic PP bands (∼ 993, 1070, and 1165 cm^–1^), which gradually shifted to those of orthoP over the course of 53 h. This was detected through the growth of the P–O stretching band of orthoP (1030 cm^–1^) and the concurrent loss (993 cm^–1^) and redshift (1165 cm^–1^ → 1100 cm^–1^) of the other PP bands (Figure 1a). Accordingly, XPS (Figure 2 and Tables S1–S3) showed that P/Mn ratios of the samples concomitantly increased from 0.03 to 0.11 (Table S2). Additionally, P–O–Mn/P–O–P (Table S3) ratios, which signaled the degradation of P–O–P oxygens to orthoP oxygens, increased from 1.07 to 1.68.

**Figure 1:**
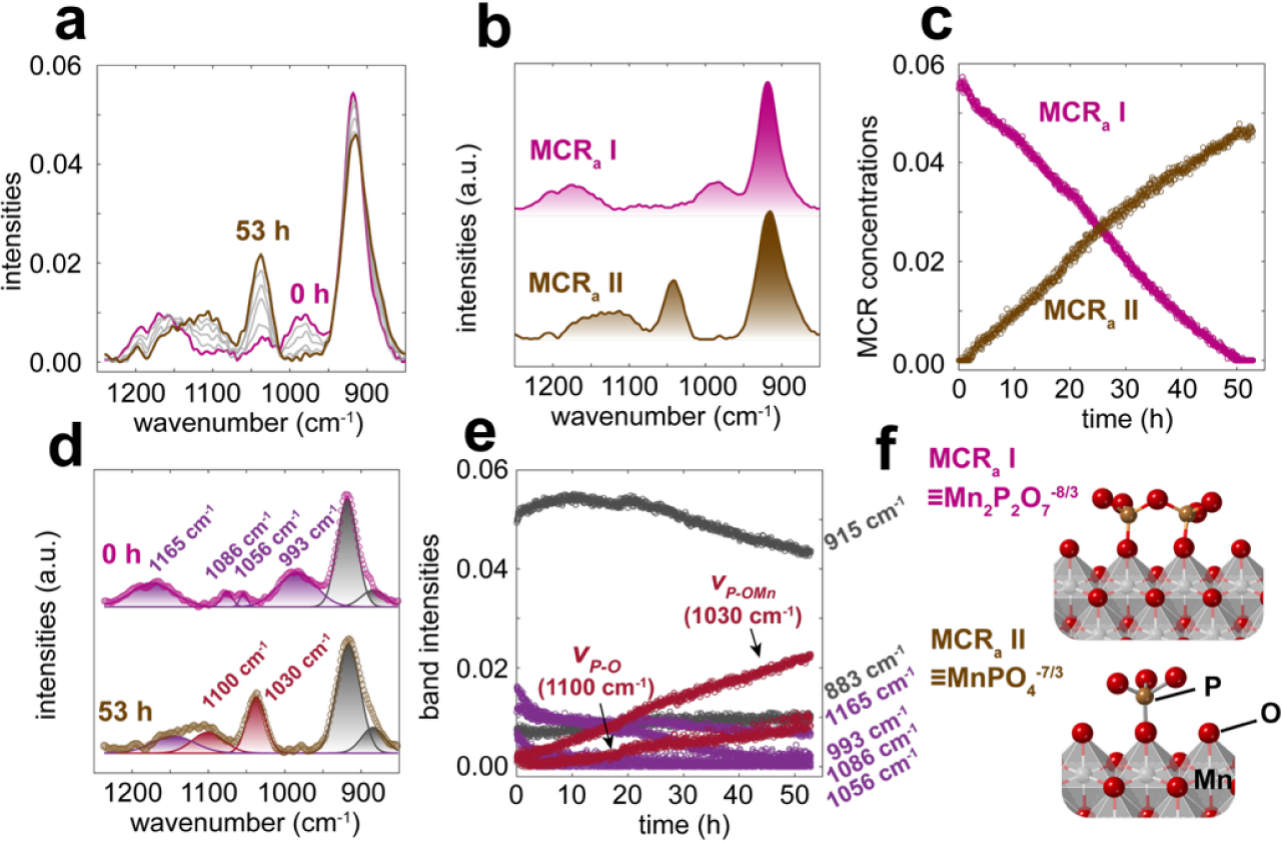
(a) ATR-FTIR spectra of PP adsorbed on MnO_2_ exposed to a flow of 80% RH N_2_(g) for 53 h. The sample was initially prepared by reacting 2 mM PP with 2.6 g/L MnO_2_ at pH 7, leading to a surface loading of *Γ* = 0.34 PP/nm^2^. MCR (b) spectral components and (c) concentration profiles from the data in (a). Gaussian deconvolution on the initial and final spectra (d) in (a), and (e) time-resolved concentration profiles. Note that the asymmetric stretching bands (900–920 cm^–1^) of the bridging P–O–P bond (ν_P–O–P,as_) of aqueous PP partly overlap with the δ_MnOH_ band (Figure S4 and Table S4).^35^ (f) Proposed surface configurations of adsorbed PP (MCR_a_ I) and orthoP (MCR_a_ II).

**Figure 2:**
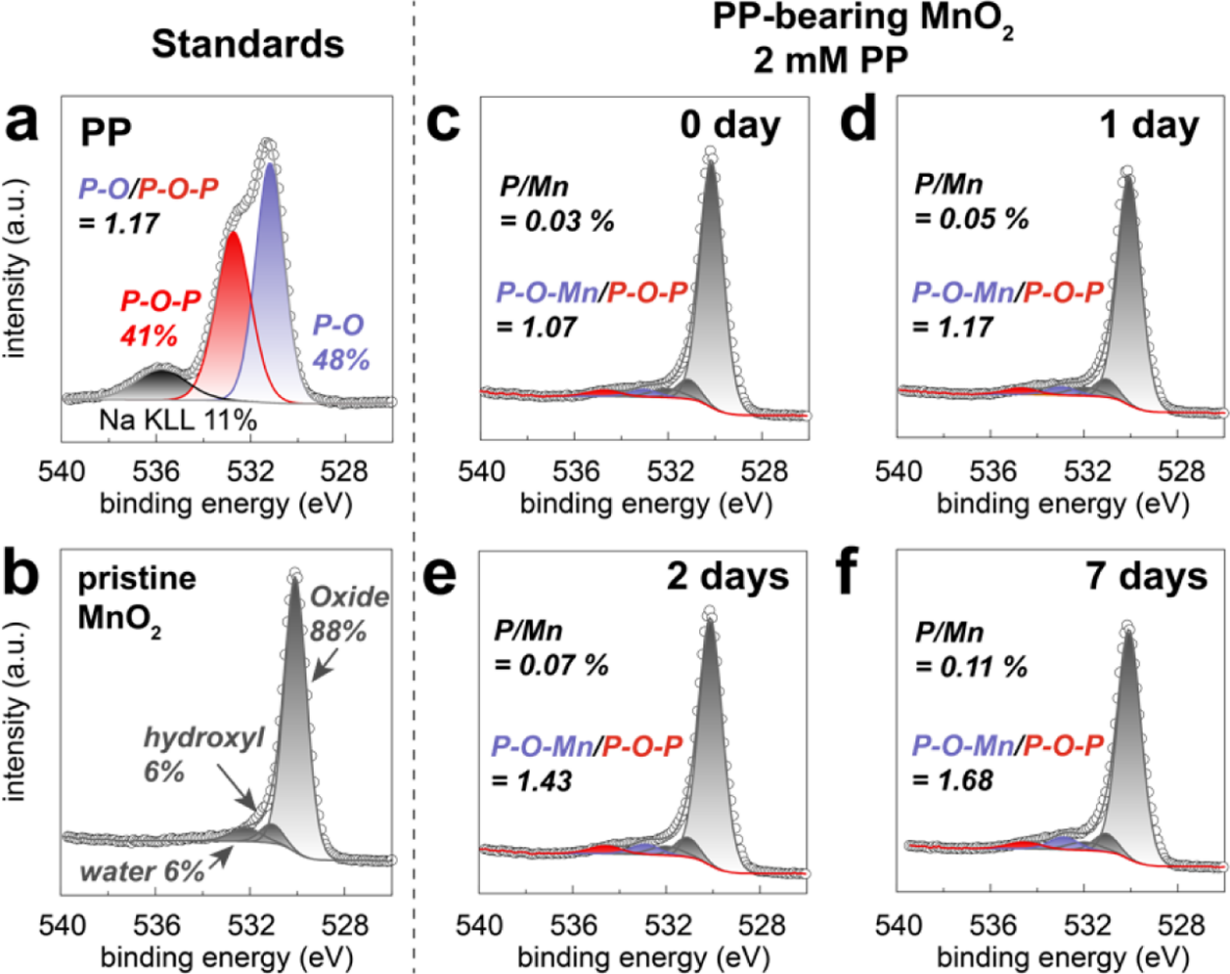
XPS spectra and fitting results of the O 1s region of (a) pure sodium pyrophosphate (Na_4_P_2_O_7_·10H_2_O), (b) pure MnO_2_, and (c-f) PP-bearing MnO_2_ (2 mM PP, pH 7) reacted for 0–7 days at 80% RH.

These transformations were also captured by two MCR_a_ vibrational spectral components, with MCR_a_ I representing PP^44^ and MCR_a_ II orthoP (Figure 1b). Their corresponding concentration profiles followed zero-order kinetics (Figure 1c), with MCR_a_ I linearly decreasing and MCR_a_ II increasing over time. A Gaussian deconvolution of MCR_a_ I resolved the four PP bands (993, 1056, 1086, ∼ 1165 cm^–1^), as well as two others (883 and 915 cm^–1^) from the bending modes of surface and nonstoichiometric Mn–OH groups (δ_MnOH_) of Mn­(OH)_2_.^45^ Spectral component MCR_a_ II was, on the other hand, deconvoluted into only two bands, one sharp (1030 cm^–1^) and the other broad (∼ 1100 cm^–1^) (Figures 1d,e and S5).

To resolve the mechanisms of decomposition, we investigated a plausible coordination geometry for the incipient surface PP on MnO_2_ in a set of loading-dependent adsorption experiments (Figure 3). We note that dried PP solutions (0 – 10 mM) showed no signals at 80% RH, suggesting no contributions from free PP species to the spectra obtained. A MCR analysis of the resulting spectra (Figure 3a) extracted only two components: (i) MCR_b_ I representing the nonreacted MnO_2_, seen through its Mn–OH bending modes (Figure 3b), and (ii) MCR_b_ II revealing surface PP species (Figure 3b). The concentrations of these components also directly scaled with PP concentrations (Figure 3c). A Gaussian deconvolution of MCR_b_ II revealed that the PP complex could be described by four comparable bands (993, 1056, 1086, ∼ 1065 cm^–1^; Figures 2d,e and Table S4) to MCR_b_ II, with their respective concentrations profiles scaling with PP concentrations (Figures 3f and S6). These results consequently revealed the existence of only one PP species on MnO_2_.

**Figure 3:**
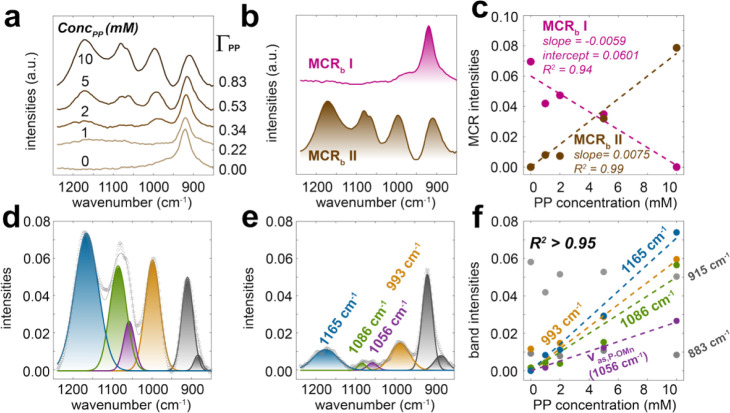
(a) ATR-FTIR spectra of adsorbed PP (Γ_PP_ in PP/nm^2^) on MnO_2_. The samples were initially prepared by reacting 1–10 mM PP with 2.6 g/L MnO_2_ at pH 7, and the spectra were obtained at 80% RH after 20 min of drying. Note that this drying time is too short for any significant PP hydrolysis (Figure 1). MCR (b) spectral components and (c) concentration profiles from the data in (a). Gaussian deconvolution of the spectra of samples reacted with (d) 10 and (e) 2 mM PP. (f) Concentration profiles of Gaussian components as a function of PP concentrations. Dashed lines are linear regressions on the PP bands.

Based on previous work,^46^ PP should adsorb under acidic to circumneutral conditions through a ligand exchange reaction with surface Mn–OH sites, forming direct P–O–Mn­(IV) bonds.^46^ We also note that, despite a difference in Mn oxidation state, the spectra of adsorbed PP were strongly comparable to the one of the aqueous Mn­(III)­PP_2_^5–^ complex.^47^ This, and other metal-PP complexes (e.g., Mg•H_2_PP), involves phosphate bidentate coordination with the metal center. It is also this coordination type that is believed to facilitate P–O–P bond cleavage^24,26,27^ by lowering the activation energies of P–O–P dissociation, producing free phosphate complexes.^48^

Given this similarity, we propose that PP surface complexes should also be in bidentate mononuclear or binuclear coordination with surface Mn sites. Although more direct information could be provided in a dedicated study (e.g., phosphorus and manganic K-edge X-ray absorption spectroscopy), numerical simulations of mineral surface-bound PP^42,46^ gave support for the binuclear coordination. Here, two terminal phosphate moieties (Figure S7) are believed to be complexed to two adjacent Mn­(IV) sites, most likely at edges of MnO_2_ nanoparticles,^49^ after ligand exchange:

3 $$2\equiv {\mathrm{MnOH}}^{-1/3}+H_{2}P_{2}{O_{7}}^{2-}⇌{\equiv \mathrm{Mn}}_{2}P_{2}{O_{7}}^{-8/3}+{2H}_{2}O$$

A slight redshift (<4 cm^–1^) in the characteristic P–O–Mn­(IV) bond (∼ 1056 cm^–1^) compared with Mn­(III)-PP complexes (∼ 1060 cm^–1^) can be explained by differences in the electronic structure of Mn­(III) and Mn­(IV) (Table S4). In accordance with previous studies,^44^ adsorbed PP was not likely to be protonated as deuteration (Figure S8) did not shift the vibrational spectra (Figure 1f, MCR_a_ I).

To further investigate the coordination environment of the orthoP species (1030 cm^–1^) formed by PP hydrolysis (Figure 1, MCR_a_ II), we acquired FTIR spectra across a range of pH and surface loadings (Figure 4). These measurements revealed the concomitant growth of a pair of bands (∼ 980 and 1150 cm^–1^) both with pH and with surface loadings. These trends were also captured by MCR (Figure 4c) through: (i) MCR_c_ I representing MnO_2_, (ii) MCR_c_ II corresponding to a low pH and low P loading orthoP species (Figure 4d), and (iii) MCR_c_ III corresponding to a high P loading orthoP species (Figure 4e). Most strikingly, MCR_c_ III contained the 1030 cm^–1^ band manifested during MnO_2_-induced PP hydrolysis (Figure 1e).

**Figure 4:**
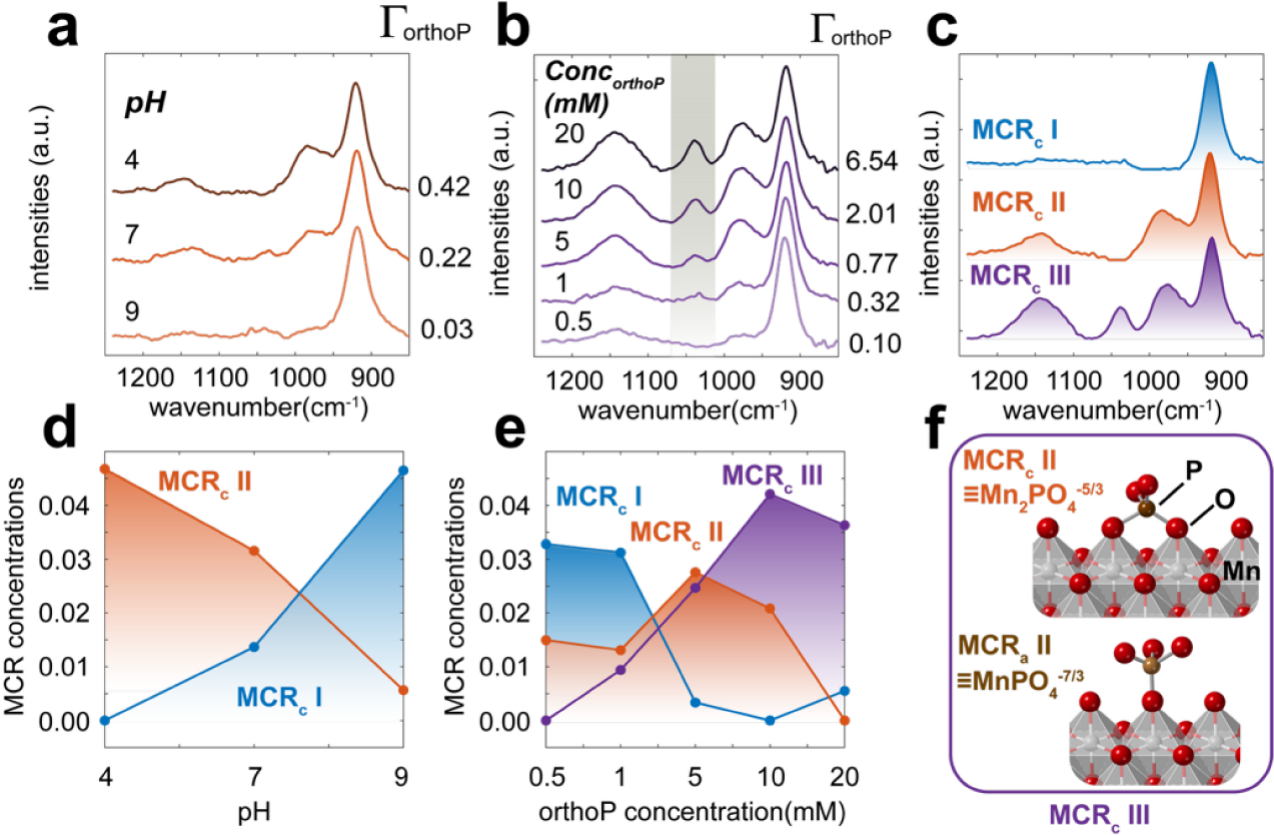
(a-b) ATR-FTIR spectra of adsorbed orthoP (Γ_orthoP_ in orthoP/nm^2^) on MnO_2_ under a stream of 80% RH N_2_(g). The samples were initially prepared by reacting suspensions of 1 g/L MnO_2_ with (a) 1 mM PP at pH 4, 7, and 9 and (b) 0.5–20 mM PP at pH 7 for 24 h in the dark. (c) MCR spectral components and (d-e) concentration profiles from the data in (a-b). (f) Proposed configurations of orthoP surface complexes extracted from MCR_c_ II and MCR_c_ III.

The appearance of this 1030 cm^–1^ band with P loadings align with previous FTIR work on orthoP binding on goethite, which is suggestive of the establishment of a single P–O–Mn­(IV) bond in a monodentate mononuclear complex of *C*_3v_ symmetry (≡MnPO_4_^–7/3^).^50,51^ We consequently propose that the remaining bands of MCR_c_ III and all the bands of MCR_c_ II, which are highly similar, are from a bidentate binuclear complex of *C*_2v_ symmetry (≡Mn_2_PO_4_^–5/3^).^14,52,53^ These collective findings therefore align with the previously established concept^46,52,54^ that low orthoP loadings favor bidentate binuclear (*C*_2v_) and high loadings monodentate mononuclear (*C*_3v_) complexes. MCR_c_ III is therefore assigned to a combination of both (Figure 4f). Additionally, as the spectra were unaffected by exposure to D_2_O (Figure S9), these complexes should be completely deprotonated.

Finally, building upon these findings, we resolved the roles that water plays in PP hydrolysis by tracking PP and orthoP spectral signatures in water films of varied loadings. This was achieved by exposing samples to moist N_2_(g) between 10 to 95% RH (Figures 5 and S10). These gases produced the equivalent of 2.6 to 7.2 monolayers (MLs) of water on PP-bearing MnO_2_ (cf. Supporting Information for details). All samples generated highly comparable spectra (Figure 5a,b), suggesting the production of the same orthoP monodentate complex regardless of air moisture.

**Figure 5:**
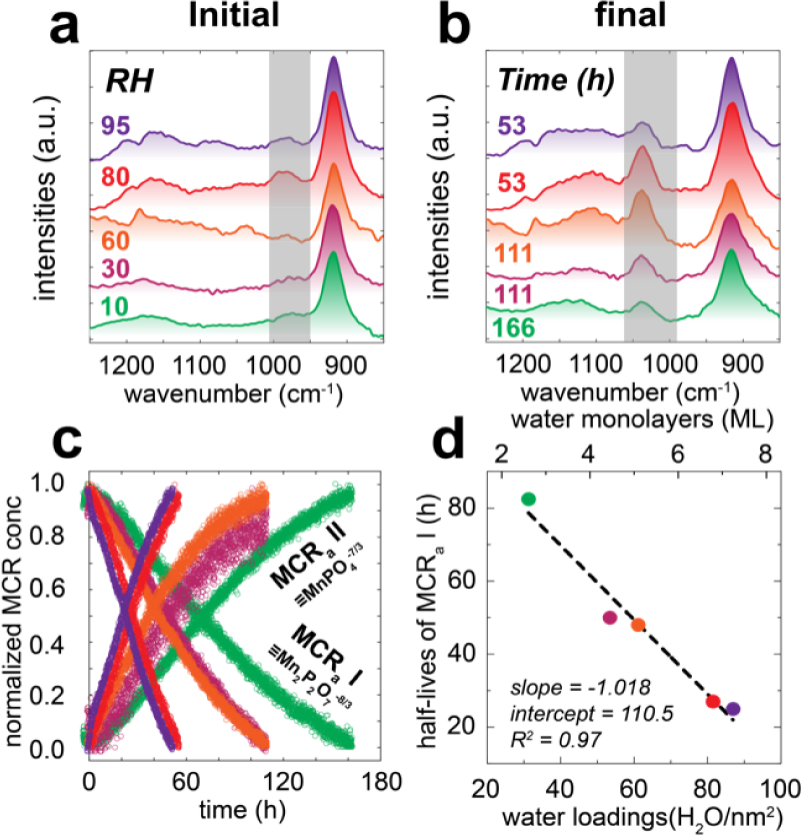
Dependence of PP hydrolysis kinetics on humidity. (a) Initial and (b) final ATR-FTIR spectra of MnO_2_ collected under a stream of 10 – 95% RH. The samples were initially prepared by reacting coatings of 2.6 g/L MnO_2_ with 2 mM PP at pH 7. (c) Color-coded normalized MCR concentration profiles of the spectra in Figure S10. MCR concentration profiles were scaled to unity. Note that the MCR components shared the same characteristics to MCR_a_ I and II in Figure 1. (d) Linear correlation between half-lives of MCR_a_ I degradation and water loadings (water monolayers).

MCR concentration profiles (Figure 5c) revealed that air moisture controlled (zero-order) PP hydrolysis rates. PP half-lives taken from these concentration profiles, decreased on the order of 12.2 h per ML, namely from 82.5 h in 2.6 ML at 10% RH down to 25 h in 7.2 ML at 95% RH (Figure 5d). This relationship thus underscored the need of available MnO_2_-bound water—including externally adsorbed and condensed water (Text S1)—in catalyzing P–O–P rupture when PP was associated with MnO_2_ under circumneutral conditions. This finding is noteworthy as previous experimental^24,26,27^ and theoretical^42,46^ studies have generally overlooked the contributions from the combined effects of mineral surface complexation and hydration on PP hydrolysis.^55,56^ Again, from the vibrational spectra, these reactions are expected to convert bidentate binuclear PP complexes (≡Mn_2_P_2_O_7_^–8/3^) into two adjacent orthoP complexes each in monodentate coordination (≡MnPO_4_^–7/3^).^42^ Direct coordination of two PP oxygens with two surface Mn atoms creates a double Lewis acid–base interaction. This activates P–O–P bonds by drawing electron density away from the P atoms and increasing P–O–P electrophilicity.^28^ Our results support the idea that, instead of surface hydroxyl groups,^22^ surface complexation increases the susceptibility of P–O–P rupture by H_2_O nucleophiles supplied by the water films. We thereby conclude that PP hydrolysis kinetics operating through the reaction:

4 $$\equiv {\mathrm{Mn}}_{2}P_{2}{O_{7}}^{-8/3}+H_{2}O⇌2\equiv {{\mathrm{MnPO}}_{4}}^{-7/3}+{2H}^{+}$$

is a zero-order process because the rate-limiting step is controlled by the availability of water molecules in the surface films, not by the concentration of PP.

## Conclusions

Polyphosphates of biogenic and anthropogenic origins tend to be degraded via a terminal-only pathway, contributing to the formation of shorter-chained counterparts, down to orthoP in nature.^57^ The interfacial behaviors, including adsorption and hydrolysis, of PP on environmentally abundant Mn oxide are of great relevance to understanding P cycling in both aquatic and humid environments.

Our *in situ* experiments showed that PP hydrolysis on MnO_2_ covered with water films obeyed zero-order kinetics with half-lives in the order of hours to days. Decomposition was driven by exposure of water and the binuclear bidentate geometry of PP at MnO_2_ surfaces. This coordination is critical for catalyzing hydrolysis reactions which produced two orthoP in monodentate mononuclear coordination. The linear dependence of PP half-lives with water loadings also revealed a significant role of surface water as a key nucleophilic agent for P–O–P bond cleavage.

This work provides molecular-level insight into the adsorption and hydrolysis of polyphosphates at mineral–water interfaces and underscores the importance of interfacial water in mediating phosphorus transformations. Notably, polyphosphate hydrolysis within nanometric water films on minerals could enable phosphorus transformation and mobilization in arid/humid environments through moisture scavenging^58^ while supporting biological activity through enhanced nutrient availability. Given that PP represents the final reactive intermediate in polyphosphate degradation, this work also informs broader questions related to phosphorus sequestration and burial^59,60^ in water-unsaturated environments. Beyond environmental implications, these findings can benefit redox biogeochemistry,^49^ catalysis,^61,62^ and energy storage solutions.^63^

## Supplementary Material



## Notes

Water sorption isotherm and changes in water vibrations upon hydration; linear correlations between DVS measurements and water vibrations; XRD patterns of MnO_2_; ATR-FTIR spectra and band assignments of aqueous orthoP and PP; band positions in Gaussian deconvolution; calculated aqueous orthoP and PP speciation; spectral response in D_2_O experiments; time-resolved ATR-FTIR spectra in humidity experiments; fitting parameters and results from XPS measurements; band assignments for P–O­(H) and Mn–O vibrations; adsorption isotherm modeling of DVS data (PDF)
