# Supplementary material for: Water Film-Mediated Hydrolysis of Pyrophosphate on a Nanosized Mn Oxide
Source: Langmuir. 2025 Aug 29;41(36):24336–43. doi: 10.1021/acs.langmuir.5c02476 (PMC12444992; doi:10.1021/acs.langmuir.5c02476)
Supplement: Supplementary file 1 [file la5c02476_si_001.pdf]

SUPPLEMENTARY MATERIALS

Water film-mediated hydrolysis of pyrophosphate on a nanosized Mn oxide

Tao Chen[1,*], Tao Luo[1], Tra My Bui Thi[2], Khalil Hanna[2], Jean-François Boily[1]

[1] Department of Chemistry, Umeå University, SE-901 87, Umeå, Sweden
[2] Univ. Rennes, École Nationale Supérieure de Chimie de Rennes, CNRS, ISCR-UMR 6226, F-35000 Rennes, France

**corresponding author: Tao Chen (tao.chen@umu.se)*

Table of Contents

Text S1 .... S2
Figure S1. .... S3
Figure S2. .... S4
Figure S3. .... S5
Figure S4. .... S6
Figure S5. .... S7
Figure S6. .... S8
Figure S7. .... S9
Figure S8. .... S10
Figure S9. .... S11
Figure S10. .... S12
Table S1. .... S13
Table S2. .... S14
Table S3. .... S15
Table S4. .... S16
Table S5. .... S17
Table S6. .... S18
References S19

Text S1

Water sorption isotherm

The water vapor sorption isotherm was modeled in terms of *adsorption* (left-term) and *condensation* (right-term) regimes, using the following expression:[1]

$$C_{\mu} = S_{ads}\frac{K_f \sum_{1}^{n=\beta+1} n \times (RH/100)^n}{1 + K_f \sum_{1}^{n=\beta+1}(RH/100)^n} + C_{\mu s}\frac{K_{\mu} \sum_{1}^{n=\alpha+1}(RH/100)^n}{K_{\mu} \sum_{1}^{n=\alpha+1}(RH/100)^n + \sum_{1}^{n=\alpha+1}(RH/100)^{n-\alpha}}$$

This was achieved by co-optimizing adjustable binding site densities for adsorbed water (S_{ads}), and condensed water ($C_{\mu s}$) with their corresponding water binding constant (K_f, K_{μ}) and bonding order (α = adjustable number of condensable water molecules; β = 2 hydrogen bonds per bound water). A non-linear least square optimization routine was used to find the best-fitting combination of parameters (Fig. S1a, Table S6) using a code written in MATLAB (The Mathworks).

Supplementary Discussion

From microgravimetry (Fig. S1a) we find that MnO_2 can be covered up to 92.5 H_2O/nm^2 (7.7 ML) at 91 % RH. The majority of surface water was via adsorption to external surface sites (*adsorption*), accounting for up to 51 H_2O/nm^2 (4.2 ML) below 60 % RH. Condensed water in multilayer sorption via hydrogen bonding on and between particles (*condensation*) was estimated as 31.7 H_2O/nm^2 (2.6 ML) at 91 % RH. The contribution from intercalated water at MnO_2 interlayers was ruled out due to the negligible changes in *d*-spacing on basal facets (d_{001}) before and during reaction (Fig. S3).

As the MCR concentrations of the water O-H stretching and bending regions (Figs. S1b-c) were linear with respect to water loadings (Fig. S2), we used this relationship to estimate water loadings in PP-bearing MnO_2 (Figs. S1d-e). These efforts revealed that PP had a slight increase in the hydrophilicity of the samples at low humidity, yet it had no effect at high humidity (Fig. S1a). For example, a water loading of 31.3 H_2O/nm^2 (2.6 ML) was observed at 10 % RH on PP-bearing MnO_2 (2 mM PP) but 22.3 H_2O/nm^2 (1.9 ML) in the absence of PP. The d_{001} showed no response to PP adsorption or degradation, suggesting no intercalation of PP or PP-derived products into MnO_2 interlayers (Fig. S3). Notably, surface-bound water, including externally adsorbed and condensed water, collectively contributed to PP degradation.

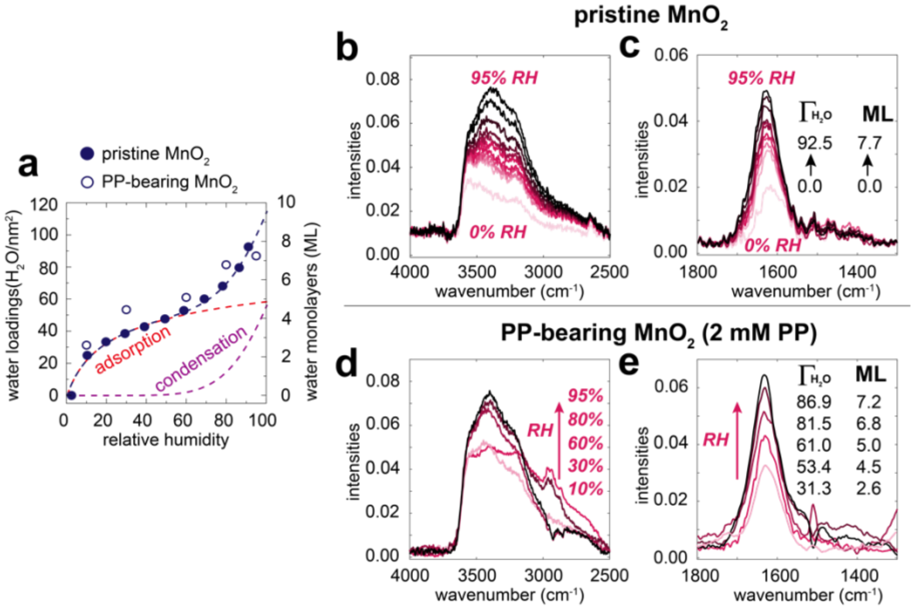

Figure S1. Water sorption on pristine and PP-loaded MnO_2 coatings. (a) Gravimetrically derived water loadings on dried MnO_2 powders and calculated water loadings on PP-bearing MnO_2 (2 mM PP, pH 7). ATR-FTIR spectra in the O-H stretching (b) and HOH bending regions (c) of N_2-dried MnO_2 coatings exposed to humid N_2 streams of 0 - 95 % RH at 25 °C. The actual water sorption amounts were calibrated using the actual spectra in O-H stretching (d) and HOH bending (e) regions of dried 2 mM PP-deposited MnO_2 coatings at pH 7 exposed to humidified N_2 at RH of 10, 30, 60, 80, and 95 % at 25 °C. Dashed lines represent model fits on pristine MnO_2 data.

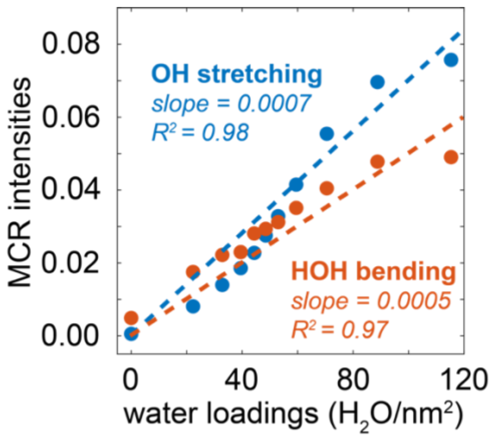

Figure S2. Linear correlations between MCR intensities at the humidity-resolved O-H stretching and H-O-H bending regions of sorbed water on pristine MnO_2 coatings (Figs. S1b-c) and water loadings obtained with DVS measurements on MnO_2 powder (Fig. S1a).

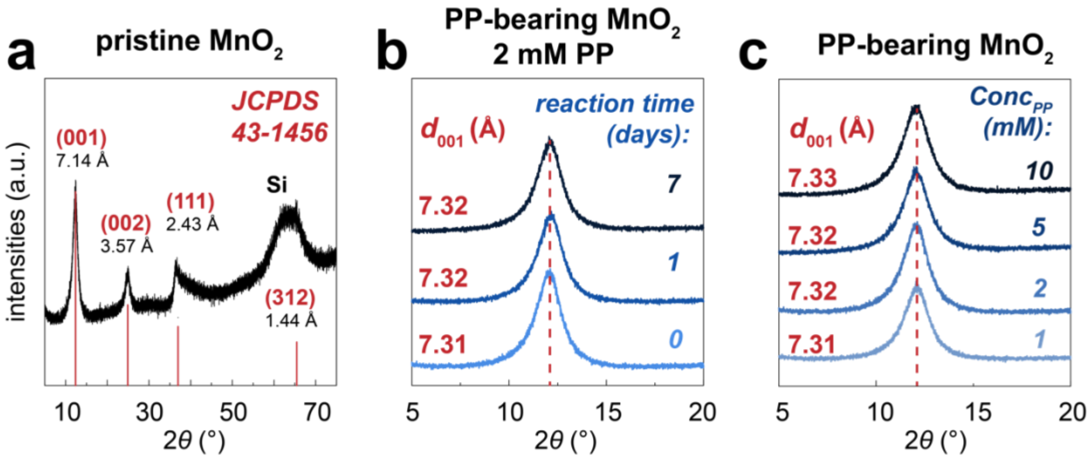

Figure S3. (a) XRD pattern of pristine MnO_2. Changes in d_{001} basal spacing of PP-loaded MnO_2 solids as a function of (b) reaction time (2 mM PP) and (c) PP concentrations (1 – 10 mM PP) at pH 7. The patterns in panel c were collected immediately after a 20-min drying. The d_{001} values are shown in Angstrom and calculated using Bragg's Law.[3] This graph shows that PP binding introduced negligible changes in d_{001}, indicating no intercalation of PP or PP hydrolysis products into MnO_2 interlayers.

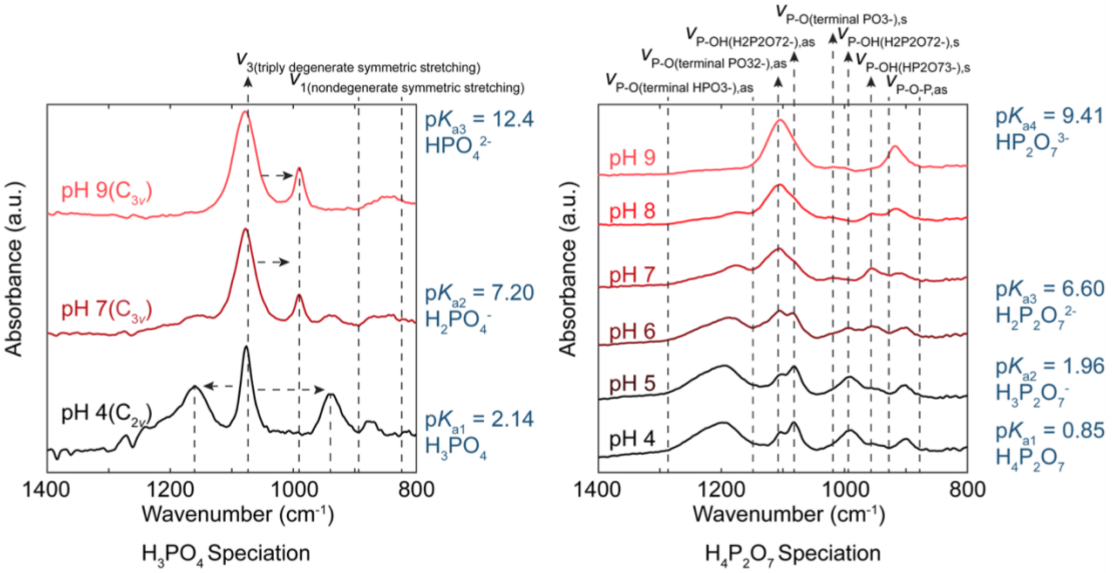

Figure S4. ATR-FTIR spectra of 50 mM solutions of orthoP (left) and PP (right) in the pH range of 4 - 9. Detailed assignments of the peaks are provided here and in Tables S4-5. The pK_a values are shown alongside for more information.

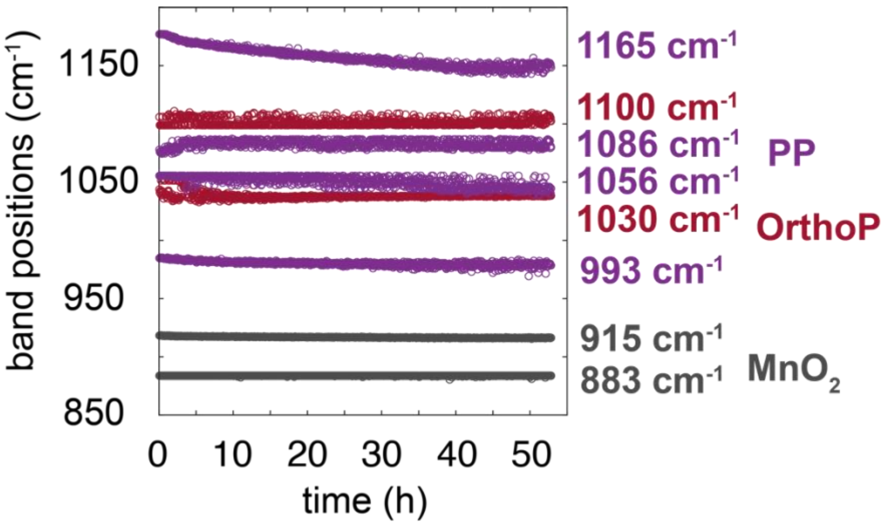

Figure S5. Color-coded band positions in the Gaussian deconvolution analysis (Figs. 1d-e) on the time-resolved pyrophosphate degradation spectra (Fig. 1a). This graph shows a stable deconvolution on the spectra with negligible fluctuations on the well-defined bands.

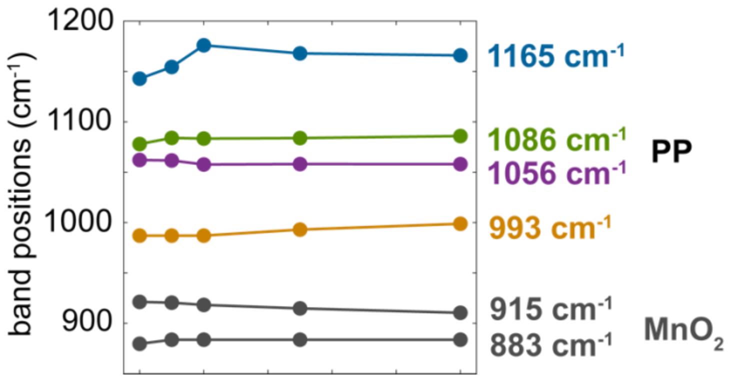

Figure S6. Color-coded band positions in the Gaussian deconvolution analysis (Figs. 3d-e) on the PP concentration-resolved spectra (Fig. 3a). This graph shows a stable deconvolution on the spectra with negligible fluctuations on the well-defined bands, except the initial point (clean MnO_2).

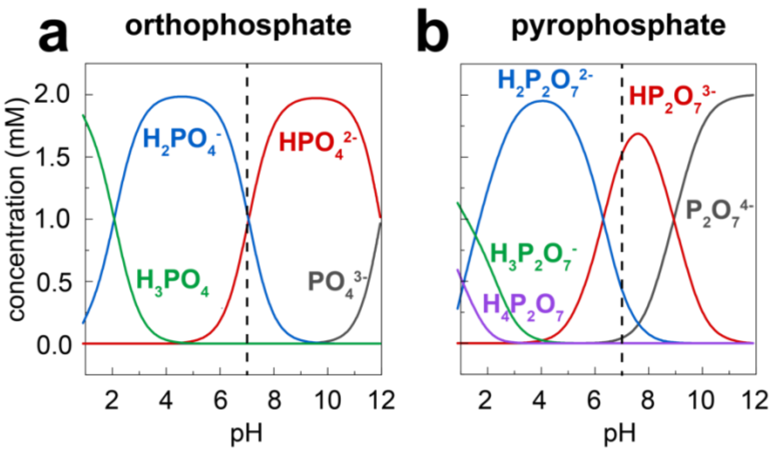

Figure S7. Aqueous speciation of 2 mM orthoP (a) and PP (b) solutions in the pH range of 1 - 12. Calculations were made using pK_a values (Fig. S4) in the MINTEQ.dat database of Phreeqc (v.3).[2]

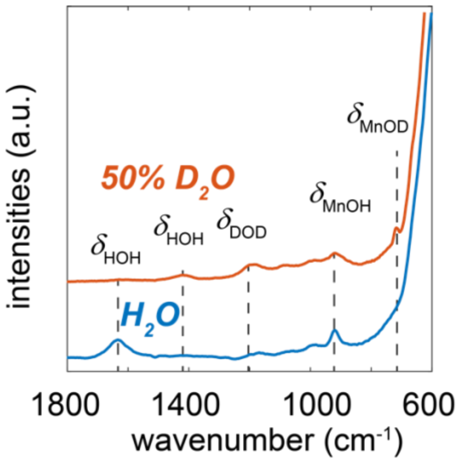

Figure S8. ATR-FTIR spectra in P-O(H) regions of PP-bearing MnO_2 (2 mM PP, pH 7) in H_2O and 50% D_2O water, respectively. The data were collected immediately after a 20-min drying period under a flow of dry N_2(g). Detailed assignments of the water vibrational bands are provided, and no shifts in PP-related bands were observed upon deuteration.

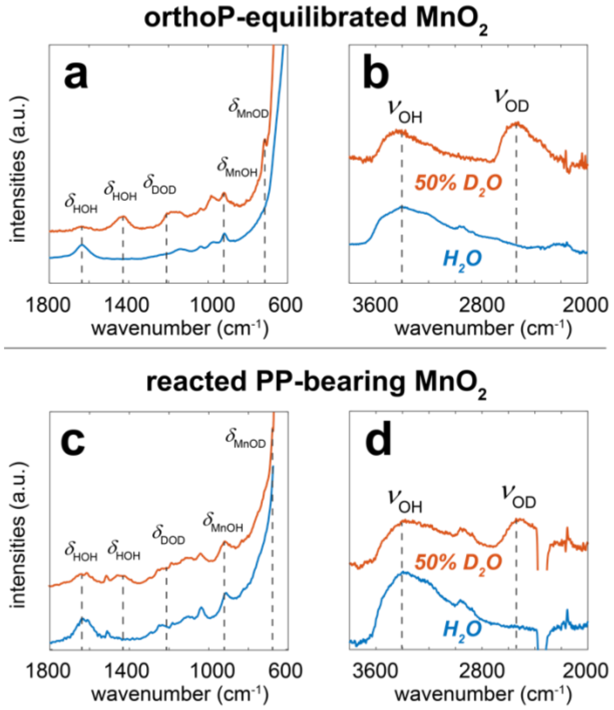

Figure S9. Vibrational spectra of the H-O-H bending and P-O(H) regions of orthoP-equilibrated MnO_2 (a, b) and reacted PP-bearing MnO_2 (c, d) in H_2O and 50 % D_2O water, respectively. The PP-bearing MnO_2 was reacted with 2 mM PP at pH 7 in 53 h of exposure to a stream of 80 % RH N_2. Detailed assignments of the water vibrational bands are provided. This graph shows no shifts in orthoP-related peaks upon deuteration.

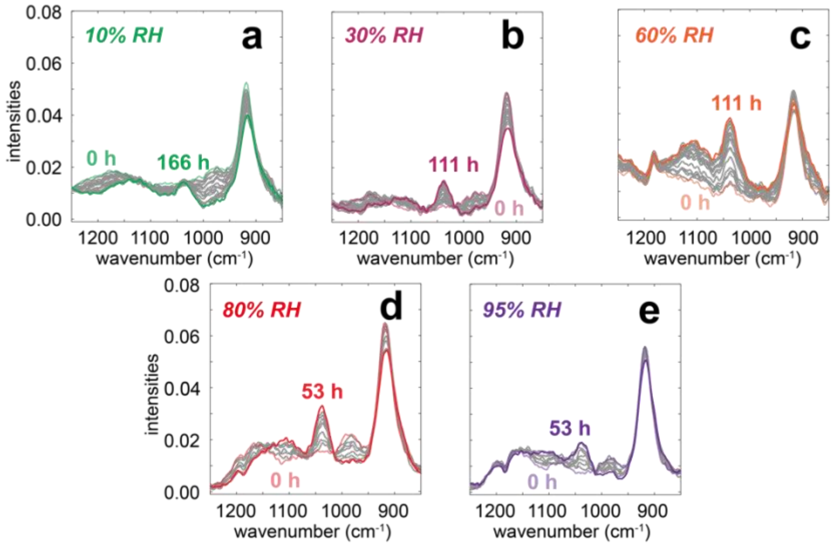

Figure S10. Time-resolved ATR-FTIR spectra in the P-O(H) regions of MnO_2 reacted with 2 mM PP at pH 7 at RH of 10 % (a), 30 % (b). 60 % (c), 80 % (d), and 95 % (e) at 25 °C.

Table S1. Fit parameters for O1s spectra of the standards and the PP-bearing MnO_2 samples (2 mM PP, pH 7).

Samples	Peak	BE (eV)	FWHM	GL	Species	Ref
Pure MnO₂	Oxide	530.14	1.11	30	O^{2-} structural	4
	Hydroxyl	531.14	1.11	30	OH^- structural	4
	Water	532.24	1.54	30	H_2O sorbed	4
Pyrophosphate	P-O	531.19	1.42	30		5
	P-O-P	532.73	1.72	30		5
	Na KLL	535.79	2.54	30		5
PP-bearing MnO₂	Oxide	530.14	1.19	30		4
	Hydroxyl	531.14	1.19	30		4
	Water	532.24	1.62	30		4
	P-O-Mn	532.91	1.43	30		6–8
	P-O-P	534.62	1.62	30		6,7

Table S2. Proportions of Mn, O, and P in the PP-bearing samples (2 mM PP, pH 7) from XPS measurements.

Samples	Mn 2p (%)	O 1s (%)	P 2p (%)	P/Mn
0 day	27.71	71.59	0.70	0.03
1 day	27.37	71.38	1.24	0.05
2 days	26.93	71.29	1.79	0.07
7 days	25.87	71.38	2.74	0.11

Table S3. Fitted results in O1s spectra of the PP-bearing samples (2 mM PP, pH 7).

Samples	Oxide	Hydroxyl	H_2O	P-O-Mn	P-O-P	P-O-Mn/P-O-P
0 day	83.29	6.66	4.16	3.04	2.84	1.07
1 day	82.58	6.6	4.13	3.64	3.06	1.17
2 days	81.91	6.55	4.09	4.38	3.06	1.43
7 days	80.92	6.47	4.04	5.37	3.20	1.68

Table S4. Assignments of FTIR peaks for aqueous and surface PP species, together with surface Mn-OH vibrations.

Species	Wavelength (cm^{-1})	Assignments	Ref
Pure MnO₂	915	δ_{MnOH}	9
	883	δ_{MnOH}	9
Free PP	1170-1197	ν_{as} (P–OH in terminal HPO_3^{2-})	10
	~1108	ν_{as} (P–O in terminal PO_3^{2-})	10
	~1084	ν_{as} (P–OH in $H_2P_2O_7^{2-}$)	10
	1009-1028	ν_s (P–O in terminal PO_3^{2-})	10
	997	ν_s (P–OH in $H_2P_2O_7^{2-}$)	10
	952-960	ν_{as} (P–OH in $HP_2O_7^{3-}$)	10
	902-920	ν_{as} (P–O–P)	10
[a] **$\equiv Mn_2P_2O_7^{-8/3}$**	~1165	ν_{as} (P=O in terminal PO_3^{3-})	10,11
	1086	ν_{as} (P–O in terminal PO_3^{3-})	10,11
	1056	ν_{as} (P–O–Mn(IV))	11
	993	ν_s (P–O in terminal PO_3^{3-})	10,11

The surface PP complexes can be described as:

[a] $2\equiv MnOH^{-1/3} + H_2P_2O_7^{2-} \rightleftharpoons \equiv Mn_2P_2O_7^{-8/3} + 2H_2O$

Table S5. Assignments of FTIR peaks for aqueous and surface orthoP species.

Species	Wavelength (cm^{-1})	Symmetry	Assignments	Ref
Free orthoP	~1159		ν_3 (triply degenerate)	12
	1077	C_{3v}	ν_3	12
	940		ν_3	12
	1078	C_{2v}	ν_3	12
	990		ν_3	12
	800-890		ν_1 (nondegenerate)	12
[a] $\equiv Mn_2PO_4^{-5/3}$	~1150	C_{2v}	ν(P-O)	13
	~980		ν(P–OMn(IV)), ν(P-O)	8,13
[b] $\equiv MnPO_4^{-7/3}$	~1100	C_{3v}	ν(P-O)	13
	1030		ν(P–OMn(IV)), ν(P-O)	13

The surface orthoP complexes can be described as:

[a] $2\equiv MnOH^{-1/3} + H_2PO_4^{-} \rightleftharpoons \equiv Mn_2PO_4^{-5/3} + 2H_2O$

[b] $\equiv MnOH^{-1/3} + HPO_4^{2-} \rightleftharpoons \equiv MnPO_4^{-7/3} + H_2O$

Table S6. Adsorption isotherm modeling parameters predicting the DVS data.

	S_{ads}(H_2O/nm^2)	K_f	β(#H_2O)	$C_{\mu s}$(H_2O/nm^2)	K_μ	α(#H_2O)
Pure MnO_2	41.3	8.91	2.0	200.0	0.395	7.05

References

(1) Do, D. D.; Do, H. D. Model for Water Adsorption in Activated Carbon. *Carbon* **2000**, *38* (5), 767–773.

(2) Lu, P.; Zhang, G.; Apps, J.; Zhu, C. Comparison of Thermodynamic Data Files for PHREEQC. *Earth-Science Rev.* **2022**, *225*, 103888.

(3) Cheng, W.; Lindholm, J.; Holmboe, M.; Luong, N. T.; Shchukarev, A.; Ilton, E. S.; Hanna, K.; Boily, J. F. Nanoscale Hydration in Layered Manganese Oxides. *Langmuir* **2021**, *37* (2), 666–674.

(4) Banerjee, D.; Nesbitt, H. W. Oxidation of Aqueous Cr(III) at Birnessite Surfaces: Constraints on Reaction Mechanism. *Geochim. Cosmochim. Acta* **1999**, *63* (11–12), 1671–1687.

(5) Gaskell, K. J.; Asunskis, A. L.; Sherwood, P. M. A. Sodium Polyphosphate ($Na_4P_4O_{12}$) by XPS. *Surf. Sci. Spectra* **2002**, *9* (1), 151–158.

(6) Sun, Z.; Zhang, N.; Cai, Y.; Kong, Q.; Yao, X.; Tian, H.; Ma, Z.; Zhang, Y.; Su, Z. Controllable Preparation of Cation Vacancy-Modulated Oxygen Defect $Mn_2^{-x}P_2O_7^{-y}$ Anodes to Enhance Lithium Storage Performance. *CrystEngComm* **2025**, No. 10, 1377–1380.

(7) Zorn, G.; Gotman, I.; Gutmanas, E. Y.; Adadi, R.; Salitra, G.; Sukenik, C. N. Surface Modification of Ti45Nb Alloy with an Alkylphosphonic Acid Self-Assembled Monolayer. *Chem. Mater.* **2005**, *17* (16), 4218–4226.

(8) Daou, T. J.; Begin-Colin, S.; Grenèche, J. M.; Thomas, F.; Derory, A.; Bernhardt, P.; Legaré, P.; Pourroy, G. Phosphate Adsorption Properties of Magnetite-Based Nanoparticles. *Chem. Mater.* **2007**, *19* (18), 4494–4505.

(9) Julien, C. M.; Massot, M.; Poinsignon, C. Lattice Vibrations of Manganese Oxides: Part I. Periodic Structures. *Spectrochim. Acta - Part A Mol. Biomol. Spectrosc.* **2004**, *60* (3), 689–700.

(10) Guan, X. H.; Liu, Q.; Chen, G. H.; Shang, C. Surface Complexation of Condensed Phosphate to Aluminum Hydroxide: An ATR-FTIR Spectroscopic Investigation. *J. Colloid Interface Sci.* **2005**, *289* (2), 319–327.

(11) Ta, H. T. T.; Tieu, A. K.; Zhu, H.; Yu, H.; Ta, T. D.; Wan, S.; Tran, N. V.; Le, H. M. Chemical Origin of Sodium Phosphate Interactions on Iron and Iron Oxide Surfaces by First Principle Calculations. *J. Phys. Chem. C* **2018**, *122* (1), 635–647.

(12) Elzinga, E. J.; Sparks, D. L. Phosphate Adsorption onto Hematite: An in Situ ATR-FTIR Investigation of the Effects of pH and Loading Level on the Mode of Phosphate Surface Complexation. *J. Colloid Interface Sci.* **2007**, *308* (1), 53–70.

(13) Tejedor-Tejedor, M. I.; Anderson, M. A. Protonation of Phosphate on the Surface of Goethite As Studied by CIR-FTIR and Electrophoretic Mobility. *Langmuir* **1990**, *6* (3), 602–611.
